# *Eucalyptus globulus* Pyroligneous Extract as Dietary Additive for Nile Tilapia Health: In Vitro and In Vivo Assessments

**DOI:** 10.3390/microorganisms13081773

**Published:** 2025-07-30

**Authors:** Marcelo Felisberto dos Reis, Nycolas Levy-Pereira, Nathalia Raissa de Alcântara Rocha, Talita Maria Lazaro, Marisa Matias de França, Sofia Harumi Lopes Nishikawa, Silvia Helena Seraphin de Godoy, Ricardo Luiz Moro de Sousa

**Affiliations:** Laboratory of Zootechnical Hygiene, Faculty of Animal Science and Food Engineering (FZEA), University of São Paulo (USP), Pirassununga 13635-900, SP, Brazil; marcelofreis5@gmail.com (M.F.d.R.); nalcantara@usp.br (N.R.d.A.R.); talita.mlazaro@usp.br (T.M.L.); marisa.franca18@gmail.com (M.M.d.F.); sofia.nishikawa@usp.br (S.H.L.N.); silviaseraphin@usp.br (S.H.S.d.G.); rlmoros@usp.br (R.L.M.d.S.)

**Keywords:** pyroligneous extracts, immunostimulants, aquaculture, *Eucalyptus globulus*, fish health

## Abstract

Studies on plant extracts as growth promoters and immunostimulants have shown promising results. However, their effects on fish health and growth remain unclear. This study evaluated the in vitro and in vivo effects of *Eucalyptus globulus* pyroligneous extract (PE) on Nile tilapia. In vitro, minimal inhibitory and bactericidal concentration (MIC and MBC) and antibiogram analyses showed that PE could eliminate key bacterial strains affecting fish and human health, but only if its volatile components were preserved. In vivo, *Oreochromis niloticus* juveniles were fed diets containing 0.5% and 1% PE. We assessed fish hematology, phagocytosis, survival against *Streptococcus agalactiae*, and growth parameters. Fish fed 1% PE had lower erythrocyte and lymphocyte counts but higher neutrophil levels than controls. Their phagocytic capacity was significantly enhanced compared to both the control and 0.5% groups. However, the 0.5% PE group had a higher phagocytic index than both the control and 1% groups. No protection against *S. agalactiae* or significant effects on growth were observed. In conclusion, distilled *E. globulus* PE shows potential as an immunostimulant for fish. However, further studies are needed to preserve its volatile compounds and optimize its use in aquaculture.

## 1. Introduction

Global aquaculture production has constantly increased in recent decades due to the depletion of natural fish stocks, escalating global population growth fueling higher seafood consumption, and rising demand for healthier dietary choices [1]. As aquaculture intensifies, fish are increasingly subjected to stress, leading to more frequent and severe infectious disease outbreaks, resulting in substantial mortality and economic losses [2,3]. To combat disease outbreaks, many producers rely heavily on antibiotics, a practice linked to detrimental effects such as toxicity, immunosuppression, and bacterial resistance [4,5,6,7,8]. Consequently, there has been a shift towards non-antibiotic dietary additives. Several studies have demonstrated the potential of alternatives such as prebiotics [9,10], probiotics [11,12,13], and particularly plant extracts [14,15].

In particular, plant extracts present a diverse array of molecules with varied chemical properties, and are extensively utilized across the food, hygiene, and pharmaceutical industries [16,17,18]. Numerous studies highlight the advantageous effects of plant extracts on fish, particularly as immunostimulants and growth enhancers [15,19,20,21]. According to the consulted literature, much of the research in aquaculture has focused on using oils or aqueous solutions extracted from fresh or dried plants [22,23,24], but no studies have investigated the effects of pyroligneous extracts (PEs) on fish health.

PE is an aqueous solution derived from the distillation of the pyrolysis liquid, a byproduct of the charcoal production process [25]. PEs from various plant species have found widespread applications across multiple sectors, serving as sterilizing agents, fertilizers, pharmaceutical ingredients, and food additives. The functional diversity of PEs can be attributed to their rich composition, which includes a variety of substances such as acids, phenols, aldehydes, ketones, alcohols, and other organic compounds [18,26]. In Brazil, PEs are predominantly produced by burning wood of *Eucalyptus* sp., a genus renowned for its medicinal properties. These trees contain beneficial organic compounds like eucalyptol, globulol, and caryophyllene oxide [27]. Some studies have found beneficial results of *Eucalyptus* sp. derivatives for aquaculture such as immunostimulation, observed in Cyprino carpio [21,28], and antibacterial properties, for exemple, against *Aeromonas caviae* [19], *Vibrio alginolyticus*, and *Vibrio harveyi* [29,30]. Despite the promising properties of Eucalyptus PEs, the consulted literature contains few studies evaluating their effects on aquaculture-relevant bacteria, and none have assessed their impact on fish health through in vivo experimentation.

*E. globulus* is among the most extensively studied species within the genus, recognized for its potent antibacterial and antifungal properties, as well as for its widespread availability in Brazil [31,32,33,34]. Nile tilapia (*Oreochromis niloticus*) is one of the most important fish species for world freshwater aquaculture, being cultivated in 126 countries [35], and responsible for more than 60% of Brazilian aquaculture production [36].

Thus, the aim of this study was to investigate the effects of *E. globulus* PE on bacteria relevant to tilapia culture, and to assess the impact of dietary inclusion of this PE on the hematology, phagocytosis, survival to bacterial challenges, and growth parameters of Nile tilapia *Oreochromis niloticus*.

## 2. Materials and Methods

### 2.1. Ethics

All the experimental procedures were performed in accordance with the National Council of Animal Experimentation Control (CEBEA) and Commission for Animal Use Ethics (CEUA) (protocol #9321250521).

### 2.2. MIC, MBC, and Antibiogram

The PE used in this study was kindly donated by AG Toniato Indústria, Comércio e Serviços LTDA (Dois Córregos, São Paulo, Brazil). The antimicrobial efficacy of PE was assessed through the standard broth dilution method, evaluating both the minimal inhibitory concentration (MIC) and minimal bactericidal concentration (MBC) across six bacterial strains (Table 1). For the MIC assessment, PE was serially diluted in two-fold increments, ranging from the pure extract to a concentration of 1 µL mL^−1^ (1:1 to 1:512 *v*/*v*) in BHI broth (Brain Heart Infusion, Oxoid, UK) within a 96-well round-bottomed plate. Subsequently, an equal volume of Brain Heart Infusion (BHI) broth containing 1 × 10^8^ CFU mL^−1^ (0.10 OD at 625 nm) was added to each well. Chloramphenicol at 2.5 mg mL^−1^ served as the positive control, and a well containing neither PE nor chloramphenicol was used as the negative control. Chloramphenicol was included as a positive control due to its broad-spectrum activity, its widespread use in antimicrobial susceptibility testing in aquaculture [37], and the lack of reports on resistant strains in Brazil, according to the consulted literature. Following this setup, the plate was incubated for 24 h at 37 °C. Although the bacterial strains originate from aquatic environments, incubation at 37 °C was chosen because it is the standard temperature for in vitro antimicrobial susceptibility protocols, ensuring assay standardization and reproducibility. Additionally, 37 °C promotes faster and optimal bacterial growth—as evidenced by studies demonstrating efficient *A. hydrophila* proliferation at this temperature compared to 4 °C and 28 °C—thereby reducing incubation times and accelerating laboratory workflows [38]. After the incubation period, 40 µL of iodonitrotetrazolium chloride solution (2 mg mL^−1^, Sigma, Aachen, Germany) was added to each well, and after an additional 3 h incubation, the plate’s readings were taken, noting the lowest concentrations of PE that inhibited bacterial growth. Following the MIC determination of PE, 50 μL aliquots from each tube showing no visible bacterial growth were transferred to BHI agar plates. These plates were then incubated for 24 h at 37 °C to assess the MBC. After the incubation, the MBC was determined by observing the presence or absence of bacterial growth on the agar plates.

For the antibiogram assay, strains of the bacteria mentioned earlier were reactivated by adding 100 µL of bacterial suspension to 9 mL of BHI broth. This mixture was incubated at 35 °C for 24 h. Sterile antibiotic testing discs were then immersed in concentrations of 100%, 50%, 25%, 12.5%, 6.25%, and 3.12% based on prior assessments. After soaking for 30 min, the discs were transferred to an incubator at 35 °C to dry for 60 min. Next, 150 × 25 mm Petri dishes filled with Mueller–Hinton agar (Kasvi, Sao Jose dos Pinhais, Brazil) were seeded with the bacteria and the soaked discs were placed on the agar. A control disc containing 30 µg of chloramphenicol was also used. The plates were then incubated at 35 °C for 24 h.

### 2.3. Fish, Feeds, and Experimental Conditions

The experiment was conducted at the Ecopeixe fish farming Research and Development Department in Pirassununga, São Paulo, Brazil. Ecopeixe kindly donated *O. niloticus* fingerlings (0.5 ± 0.2 g), which were kept in 320 L polystyrene tanks for acclimation. After a one-month acclimation period, 270 juveniles were measured and weighed (12.7 ± 4.27 g; 8.81 ± 2.16 cm) and subsequently distributed into nine 320 L polystyrene tanks, with 30 fish per tank. Throughout the acclimation and experimental periods, 25% of the water in each tank was renewed daily to remove feces and uneaten feed and to maintain optimal water quality. The water temperature was maintained at 28 °C (±1 °C) through the use of a heater, and constant aeration was provided through aquarium pumps. Water quality parameters, including temperature (27.1 ± 0.8 °C), dissolved oxygen (4.2 ± 0.7 g/dL), and pH (7.1 ± 0.4), were monitored weekly during both the acclimation and experimental phases.

During the initial two weeks of acclimation, the fish were fed ground feed, transitioning to 0.8 mm pelleted feed for the remaining two weeks (both containing 45% crude protein, provided by Socil Animal Nutrition, Brasópolis, Brazil). They were fed twice a day until they reached apparent satiety (no fish eating at the time).

For the experiment, commercial fish feed (Socil, 1.3 mm pellets, 40% crude protein) was used to better approximate real farm conditions. The incorporation of PE into the feed followed the method described by Dairiki et al. [39], and the inclusion levels were based on Baba et al. [40] and Brum et al. [41].

Briefly, two ethanolic solutions were prepared by diluting PE at 1:10 and 1:20 (*v*/*v*) ratios. These solutions were applied to the commercial feed using a spray bottle, at a ratio of 1:10 (*v*/*w*), resulting in two treatment diets containing 1.0% and 0.5% PE, respectively. For the control group, feed was sprayed with ethanol only, using the same proportion (1:10 *v*/*w*). All feeds were air-dried in the shade, and then supplemented with 2% soybean oil to prevent PE leaching into the water. The diets were stored under refrigeration (−18 °C) until use.

The nine tanks were randomly assigned to three groups, with three tanks per group, each receiving a different experimental feed: control, 0.5% PE, and 1% PE. The concentrations of pyroligneous extract (0.5% and 1.0%) were selected based on preliminary in vitro assays that confirmed its antimicrobial activity (MIC: 3.12–6.25%; MBC: 12.5%), and adapted to in vivo conditions considering safety and palatability. Similar concentrations have previously been used for phytogenic additives in aquaculture diets [42]. Fish were fed the experimental feeds twice daily until apparent satiety was reached. To determine feed consumption accurately, each tank was equipped with its own feed bucket. The buckets were weighed before and after each feeding session, and the difference in weight was recorded to measure the amount of feed consumed.

### 2.4. Hematology

After 30 days of experimentation, 10 fish from each tank were captured and anesthetized using a eugenol solution (0.1 mL L^−1^ of water). Blood samples were then collected through veno-caudal puncture using a 3 mL syringe coated with heparin (diluted 1:50 in saline solution). Following blood collection, the fish were removed from the experiment and transferred to another tank. The collected blood was analyzed for hematological parameters according to the methods described by Paiva et al. [43]. For red blood cell (RBC) determination, 10 μL of blood was diluted in 2 mL of formaldehyde–citrate solution (Na_3_C_6_H_5_O_7_ 112 mM, CH_2_O 517 mM in Milli-Q water) and RBCs were counted using a Neubauer chamber. To prepare blood smears, 4.5 μL of heparinized blood was spread on glass slides, air-dried, and stained with May–Grünwald–Giemsa–Wright stain. Subsequently, a total count of thrombocytes and leukocytes was performed by examining 2000 erythrocytes across several fields of the smear, and a differential leukocyte count was conducted by counting 200 leukocytes.

### 2.5. Phagocytosis Assay

The in vivo phagocytosis assay was conducted as described by Levy-Pereira et al., [44]. Briefly, 1.5 g of *Saccharomyces cerevisiae* (Fleishman, Sorocaba, Brazil) was mixed with 5 mL of Phosphate Buffered Saline (PBS) (NaCl 0.137 M, KCl 2.7 mM, KH_2_PO_4_ 1.5 mM, Na_2_HPO_4_ 8.1 mM, CaCl_2_ 0.9 mM, MgCl_2_ 0.49 mM in Milli-Q water, pH 7.4) containing 0.83% Congo red. This mixture was placed in a 15 mL Falcon tube and left to stain for 15 min. Subsequently, 7 mL of Milli-Q water was added and the solution was autoclaved for 15 min. The yeast cells were then washed by centrifugation (250× *g* for 5 min). After each centrifugation, the supernatant was eliminated and autoclaved PBS was added to the yeast pellet up to 10 mL. This washing step was repeated until all excess dye was removed. Finally, the yeast cells were resuspended in autoclaved PBS and stored at 4 °C until needed. Prior to injection, the yeast concentration was adjusted to 2.5 × 10^6^ cells per μL.

After 30 days of experimentation, two fish from each tank (six fish per treatment) were anesthetized with a 0.1 mL L^−1^ eugenol solution and placed in separate aquaria. Each group of fish then received a 0.1 mL injection of the yeast solution into the coelomic cavity beneath the left pectoral fin, using 1 mL syringes fitted with 29 Gauge insulin needles.

Two hours post-injection, the fish were euthanized using a concentrated eugenol solution (1 mg L^−1^ of water), and a careful ventral incision was made with a bistoury. The coelomic cavity was rinsed with 600 μL of Hanks’ Balanced Salt Solution (HBSS) (137 mM NaCl, 5.4 mM KCl, 0.25 mM Na_2_HPO_4_, 0.44 mM KH_2_PO_4_, 1.3 mM CaCl_2_, 1 mM MgSO_4_, 4.2 mM NaHCO_3_) enriched with 100 IU heparin using a 200 μL micropipette (Eppendorf, Hamburg, Germany). The cell suspension was collected in a 1.5 mL V-bottomed Eppendorf tube and centrifuged at 250× *g* for 5 min at 4 °C, then kept on ice until microscopy analysis.

The supernatant was discarded, and the pellet was placed between a glass slide and coverslip. Observations were made under 400× magnification using an optical microscope (Eclipse, Ci, Nikon NI, Tokyo, Japan) equipped with a CCD camera (DS Fi1, Nikon NI, Tokyo, Japan). Phagocytic Capacity (PC = number of phagocytizing leukocytes/total number of leukocytes × 100) and Phagocytic Index (PI = number of yeast cells/number of phagocytizing leukocytes) were then calculated.

### 2.6. Zootechnical Parameters

At the end of the experiment, the zootechnical parameters were determined in order to evaluate the effects of dietary PE. For this, fish were measured and weighed at the beginning and at the end of the experiment. In order to determine the apparent feed consumption, the feed was prepared and stored in plastic containers, one for each fish tank. Fish were fed twice a day until apparent satiation and each container was weighed before and after each feeding time, and the wight difference was annotated. No natural mortality was observed.

The following equations were used:

Weight gain (WG) = Final Weight (FW) − Initial Weight (IW)

Mean weight gain (MWG) = WG ÷ number of fish

Feed Conversion Ratio (FCR) = Consumed feed ÷ WG

Mean Daily Weight Gain (MDWG) = WG ÷ days of feeding

Growth = Final Length (FL) − Initial Length (IL)

K = 100 × (FW) ÷ FL^3^

### 2.7. Bacterial Challenge

For the bacterial challenge, 100 µL of *S. agalactiae* from the previously described strain was cultured in BHI (BD, EUA) broth and incubated at 37 °C for 48 h. The bacterial suspension was washed three times by centrifugation (300× *g*, 10 min, 4 °C), and the concentration was adjusted to 10^7^ CFU mL^−1^. The prepared solution was stored at 8 °C until use.

Immediately following biometry on the same day, 10 fish from each plastic tank were randomly selected and transported from the aquaculture farm to the experimental laboratory using 60 L gallons with constant aeration. The travel time did not exceed 15 min (and the stress from it will be discussed further). In the laboratory, the fish were transferred to 60 L aquaria, with three aquaria designated for fish fed with the control diet, three for fish fed with 0.5% PE, and three for fish fed with 1% PE. Additionally, three aquaria housed 10 fish each from the control group tanks as non-injected controls.

The next day, all fish were anesthetized with a clove oil solution. Fish in the injected control, 0.5%, and 1% PE groups received 100 µL of the *S. agalactiae* solution intracelomically, while the non-injected control group received an injection of PBS. Mortality was monitored over the following 14 days. Any deceased fish were promptly removed from the aquaria, which were maintained with constant aeration and temperature control at 28 ± 0.5 °C.

### 2.8. Statistics

Results are presented as the mean ± standard error. Statistical analyses were conducted using R software, version 3.4.0. Homoscedasticity and normality of the data were verified using Levene’s test and the Cramer–Von Mises test, respectively. Hematological parameters were analyzed using the Kruskal–Wallis test, followed by post-hoc comparisons with the non-parametric Tukey–Kramer test. Phagocytic parameters following bacterial challenge were analyzed using ANOVA, with subsequent mean comparisons performed using Tukey’s multiple range test. The bacterial challenge results were analyzed in Python (V 3.11.8) using the Kaplan–Meyer test and the survival curves were compared using the Log-Rank test. In all cases, α = 5%.

## 3. Results

### 3.1. MIC and MBC

The MIC and MBC results for *E. globulus* PE are detailed in Table 2. There was no variation among replicates for each bacterial strain tested. For all bacteria, the MIC and MBC values were consistently 6.25% and 12.5%, respectively, except for *P. shigelloides*, which showed a MIC of 3.12% and no detectable MBC.

### 3.2. Antibiogram

**Figure 1:**
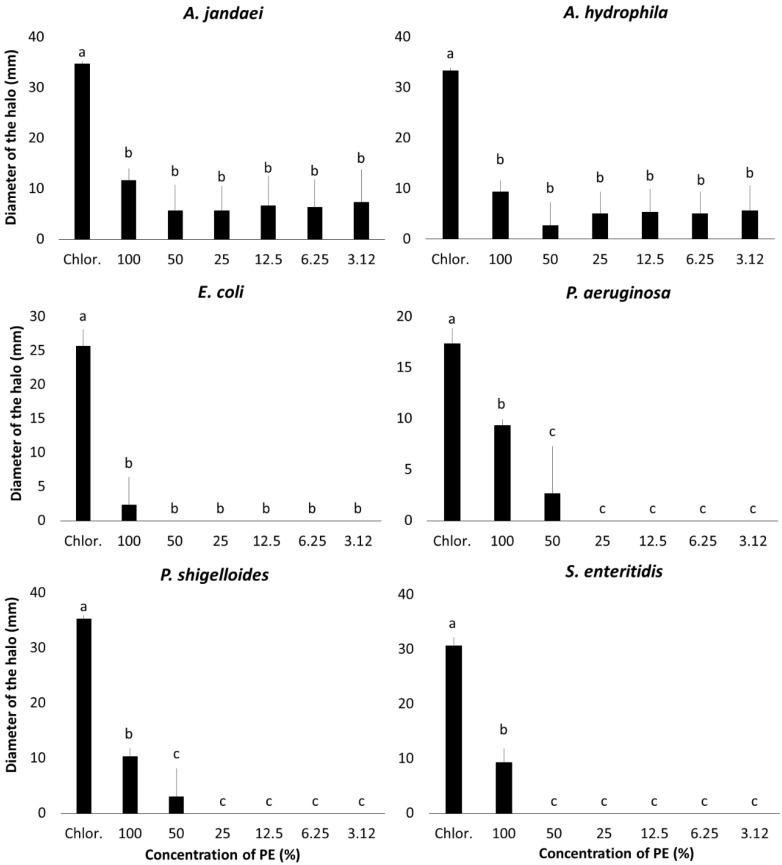
Antibiogram of *E. globulus* PE against bacteria pathogenic to fish and food contaminants. Means with different letters indicate statistical difference according to Tukey’s test (α = 5%).

The antibiogram results for PE are depicted in Figure 1, with the exception of *S. agalactiae*. For *Aeromonas jandaei* and *Aeomonas hydrophila*, no significant differences were observed across the PE dilutions, ranging from 12 ± 0.6 mm at 100% to 7 ± 6.4 mm at 3.12% for *A. jandaei* and from 9 ± 0.6 mm at 100% to 6 ± 4.9 mm at 3.12% for *A. hydrophila*, all significantly less effective than chloramphenicol (*p* < 0.0001 for both).

For *Pseudomonas aeruginosa* and *Pseudomonas shiguelloides*, concentrations of 25% or lower showed no inhibitory effect on bacterial growth. At 100% and 50%, *P. aeruginosa* exhibited inhibition zones of 9 ± 0.6 mm and *P. shiguelloides* showed 10 ± 1.5 mm and 3 ± 5.2 mm, respectively, which were statistically less effective than chloramphenicol (*p* < 0.0001 for both).

For *Escherichia coli* and *Samonella enteridis*, no inhibitory effect was observed at 50% PE or lower. At 100% concentration, PE produced an inhibition halo of 9 ± 2.5 mm for *S. enteridis*, which was significantly larger than the lower concentrations but still less than that of chloramphenicol (*p* < 0.0001). For *E. coli*, a halo of 2 ± 4 mm at 100% concentration showed no significant differences compared to other concentrations and was statistically less effective than chloramphenicol (*p* < 0.0001).

No inhibitory effect was observed for any PE concentrations against *S. agalactiae*. A second ANOVA was conducted excluding the chloramphenicol results to assess differences between the PE treatments for each bacteria strain, but it revealed no significant differences.

### 3.3. Hematological Profile

**Figure 2:**
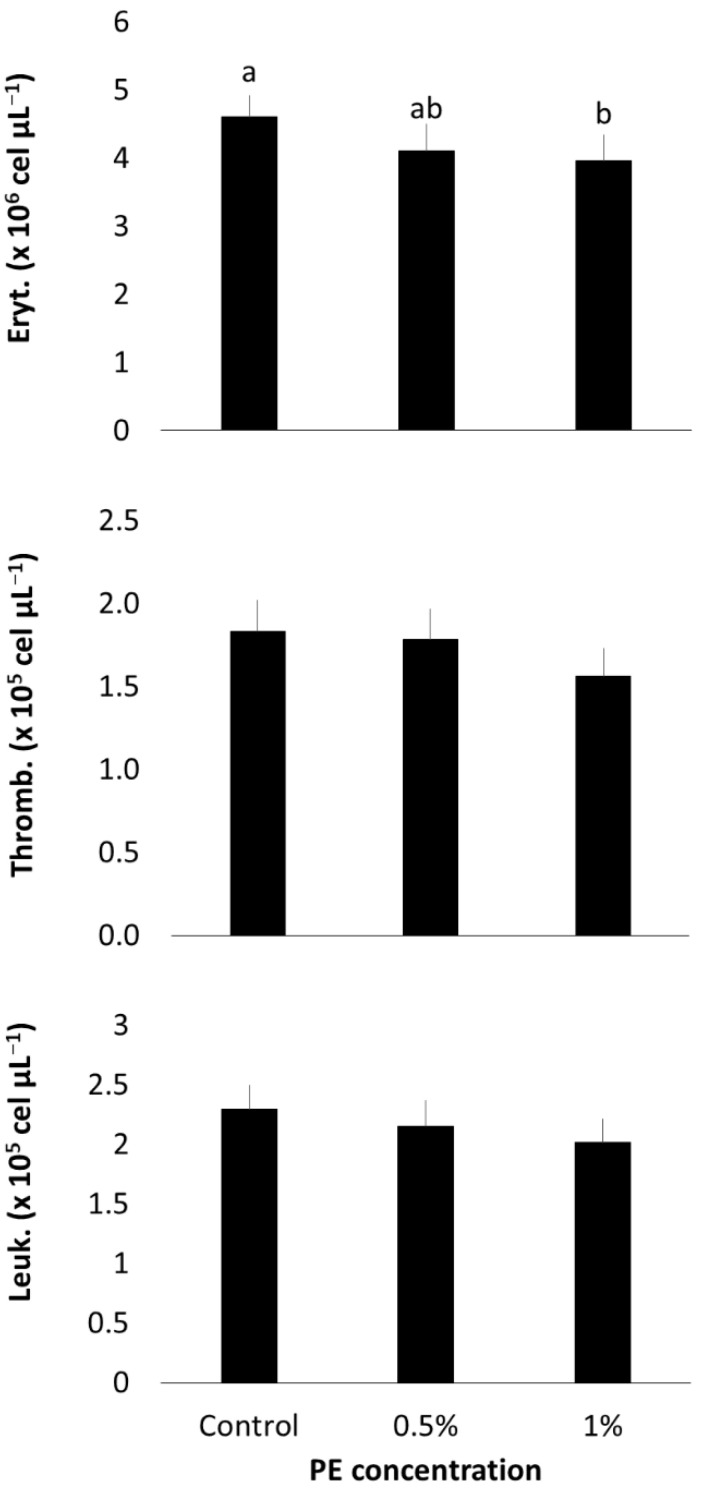
Hematological parameters of *O. niloticus* after 30 days of 0.5 and 1% of *E. globulus* PE feeding. Eryt: erythrocytes; Thromb: thrombocytes; Leuk: total leukocytes. Means with different letters indicate statistical difference according to Tukey’s test (α = 5%).

After 30 days of the experiment, fish fed with 1% PE exhibited significantly lower erythrocyte counts compared to the control group (*p* = 0.0288) (Figure 2). However, there were no significant differences between fish fed with 0.5% PE, 1% PE, and the control group. Thrombocyte and total leukocyte counts did not show significant differences across treatments (*p* > 0.05).

**Figure 3:**
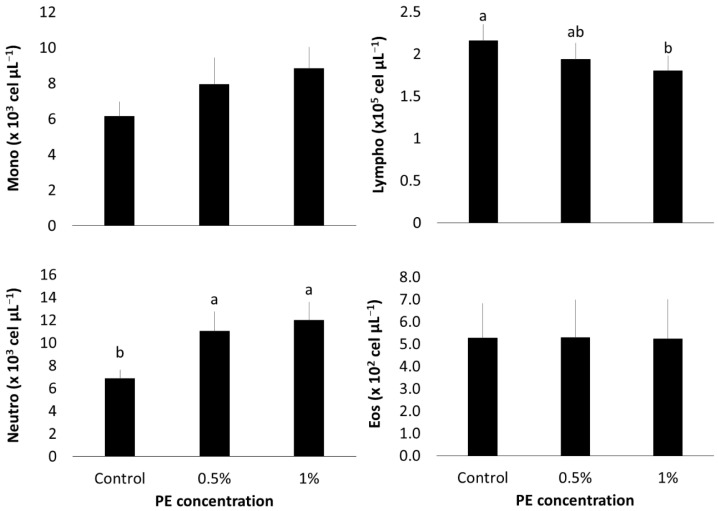
Hematological parameters of *O. niloticus* after 30 days of 0.5 and 1% of *E. globulus* PE feeding. Mono: monocytes; Lympho: lymphocytes; Neutro: neutrocytes; Eos: eosinophil. Means with different letters indicate statistical difference according to Tukey’s test (α = 5%).

Lymphocyte counts were significantly lower in the group fed with 1% PE compared to the control group (*p* = 0.0263) (Figure 3), whereas the group fed with 0.5% PE displayed lymphocyte counts similar to both the control and 1% PE groups. In contrast, neutrophil counts were higher in both the 0.5% and 1% PE groups compared to the control (*p* < 0.0001). No significant differences were observed in the counts of monocytes and eosinophils across all groups (*p* > 0.05).

### 3.4. Phagocytosis 

**Figure 4:**
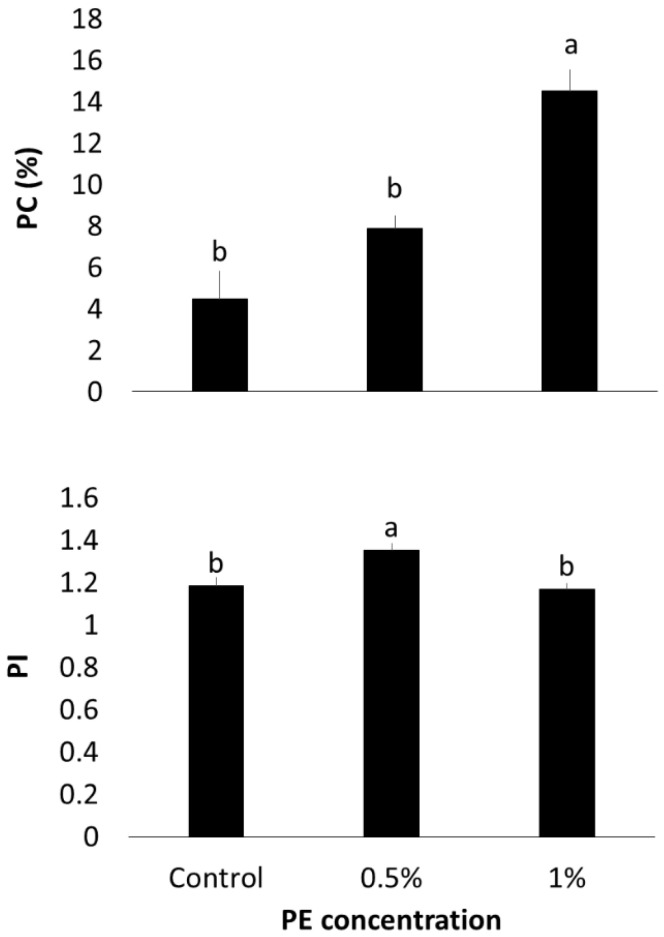
Phagocytic parameters of *O. niloticus* after 30 days of 0.5 and 1% of *E. globulus* PE feeding. PC: phagocytic capacity; PI: phagocytic index. Means with different letters indicate statistical difference according to Tukey’s test (α = 5%).

In the phagocytosis assay, the phagocytic capacity (PC) of fish fed with 1% PE was significantly higher than that observed in the 0.5% PE and control groups (*p* = 0.0002, Figure 4). Conversely, fish fed with 0.5% PE demonstrated a higher phagocytic index (PI) compared to both the control and 1% PE groups (*p* = 0.0070).

### 3.5. Zootechnical Results

No significant differences were observed after feeding *O. niloticus* with PE for 30 days (Table 3).

### 3.6. Bacterial Challenge Survival

**Figure 5:**
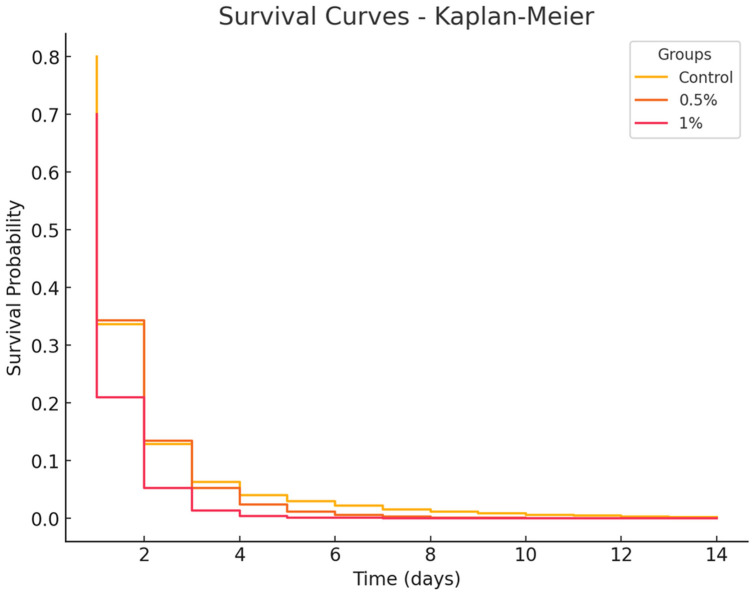
Kaplan–Meier survival analysis of *O. niloticus* fed with 0.5% and 1% *Eucalyptus globulus* pyroligneous extract for 30 days, followed by a bacterial challenge with *S. agalactiae*.

The results of the bacterial challenge with *S. agalactiae* are shown in Figure 5. After the challenge, by day 2, mortality reached 60% in the 1% group and 70% in both the control and 0.5% groups. After day 6, mortality stabilized at 74% in the 1% group, 80% in the 0.5% group, and 90% in the control group. By the end of the observation period, mortality rates were 80% in the 1% group, 83% in the 0.5% group, and 90% in the control group. However, no significant differences were observed between the treated groups or between treated groups and the control (*p* = 1.000).

## 4. Discussion

This study assessed the efficacy of *Eucalyptus globulus* pyroligneous extract (PE) both in vitro against bacterial strains relevant to the aquaculture industry and in vivo as a dietary prophylactic agent for Nile tilapia. Despite the increasing use of phytogenic compounds in aquaculture, no studies to date have explored the effects of pyroligneous extract on finfish, making this the first work to investigate both its in vitro antimicrobial activity and its in vivo immunomodulatory potential in Nile tilapia.

*E. globulus* PE showed notable bacteriostatic and bactericidal effects in MIC and MBC assays on key aquaculture bacteria, including four fish pathogens (*A. hydrophila*, *A. Jandei*, *P. aeruginosa*, *S. agalactiae*) and two general pathogens (*E. coli*, *S. enteridis*). Da Silva et al. [45] found stronger effects using *E. urograndis* PE against strains from bovine mastitis, with lower MIC and MBC values for *S. agalatiae* (0.781%), *E. coli* (1.562%), and *S. enteridis* (1.562%). Additionally, de Souza et al. [46] reported that *E. grandis* PE inhibited *C. albicans*, *S. mutans*, and *L. acidophilus* in dental biofilms by 9.98% to 100% after 24 h. These results suggest that PE composition may vary depending on the *Eucalyptus* species and pyrolysis process, which could explain the higher MIC/MBC values found in our study.

This study revealed discrepancies between antibiogram and MIC/MBC assay results using PE. While no differences were observed across dilutions for *A. jandaei* and *A. hydrophila*, there was a notable decrease in efficacy with dilution for *E. coli*, *P. aeruginosa*, *P. shiguelloides*, and *S. enteritidis*, with no effect on *S. agalactiae*. These inconsistencies are likely due to the antibiogram method, which involves drying the discs, possibly causing active compounds to volatilize, a problem absent in MIC/MBC assays where PE is applied directly to the wells.

De Souza Araújo et al. [47] reported that *E. urograndis* PE displayed larger inhibition zones against *E. coli* (20 mm) than gentamicin, the positive control (14.7 mm). They also observed inhibition zones ranging from 21.7 to 27 mm for *Pseudomonas aeruginosa*, *Staphylococcus aureus*, *Candida albicans*, and *Cryptococcus neoformans* using PE, while gentamicin showed no effect. The study further demonstrated significant antibacterial activity with inhibition halos of 10 mm at a 20% dilution. Conversely, our study found discrepancies between antibiogram and MIC/MBC results for PE. No differences were observed in PE’s effects on *A. jandaei* and *A. hydrophila* across dilutions, whereas *E. coli*, *P. aeruginosa*, *P. shiguelloides*, and *S. enteritidis* showed a rapid decline in effect with dilution, and *S. agalactiae* showed no inhibition. These discrepancies may arise from the antibiogram’s requirement to dry the discs, potentially causing loss of volatile active compounds, an issue not present in MIC/MBC assays where PE is directly applied to the wells.

In our in vivo study, we observed a dual effect of PE on Nile tilapia health, characterized by a reduction in erythrocytes and lymphocytes, accompanied by a marked increase in neutrophils and phagocytic activity.

The decrease in erythrocytes and lymphocytes suggest that dietary inclusion of *Eucalyptus globulus* PE may have triggered a mild stress response in Nile tilapia. PE derived from *Eucalyptus* species is known to be rich in acetic acid, phenols, and cresols, compounds reported to negatively affect hematopoiesis in mammals, resulting in reduced erythrocyte and leukocyte counts [48,49,50]. This hypothesis is supported by contrasting findings from studies using other *Eucalyptus* derivatives. Nurudeen et al. [51] reported increased total leukocyte and lymphocyte counts, along with decreased neutrophils, in tilapia fed with *E. globulus* leaf extract. Similarly, Hoseini et al. [52] observed elevated erythrocyte counts after feeding tilapia with diets containing 1,8-cineole, a major component of *Eucalyptus* essential oil, for 50 days.

Conversely, the increase in neutrophils and enhanced phagocytic activity in both PE-fed groups suggests a concurrent immunostimulatory effect. *Eucalyptus* extracts have been shown to improve phagocytic parameters in human macrophages in vitro [53]. However, to date, no studies have assessed phagocytosis in fish models in vivo or in vitro [22,54].

No significant effects on zootechnical parameters were observed due to PE feeding. Our results align with various studies that have utilized plant derivatives in fish diets. Nevertheless, our findings contrast with those of Immanuel et al. [55], who reported that *Cynodon dactylon*, *Aegle marmelos*, *Withania somnifera*, and *Zingiber officinale* powders (extracted with acetone) increased the growth of *Oreochromis mossambicus* by 27 to 39% when administered through feed over 45 days. A meta-analysis by Reverter et al. [56] indicated that variations in zootechnical outcomes might be influenced by several factors, including the plant species, extraction methods, characteristics of the final product, and notably, geographic location, which encompasses a broad spectrum of edaphoclimatic conditions specific to each region. This could also explain the lack of significant results in survival during the *S. agalactiae* challenge. Moreover, this supports the notion that phytogenic feed additives should be evaluated within specific regional and production contexts before broad recommendations can be made.

No protective effects of PE feeding were observed in the bacterial challenge. Although Lin et al. [57] used *Eucalyptus* essential oil rather than PE, they reported increased survival in Trachinotus ovatus fed 10 mL kg^−1^, highlighting the potential of *Eucalyptus* extracts in aquaculture. In our study, the lack of protective effects from the treatments may be attributed to two main factors. First, the drying and storage methods of the experimental feeds may have led to the loss of volatile compounds with known bacteriostatic and bactericidal activity, which are commonly described in *Eucalyptus pyroligneous* extracts. Second, the low survival rate observed in the control group (50%) suggests that the fish were likely stressed by transportation from the farm-based plastic tanks to the laboratory aquaria, despite rapid handling. Thus, it is possible that the immunomodulatory effects observed in hematological and phagocytic parameters were insufficient to confer effective protection against *Streptococcus agalactiae*, potentially due to stress-induced immunosuppression caused by the transport process prior to the bacterial challenge.

In conclusion, in vitro experiments demonstrated that *E. globulus* PE exhibited significant bacteriostatic and bactericidal effects on bacteria relevant to both fish and human health. However, our results suggest that the active compounds responsible for these effects are volatile, which should be taken into consideration for product storage as well as feed preparation and preservation. *E. globulus* PE also induced immunostimulation in *O. niloticus*, as evidenced by improvements in hematological and phagocytic parameters, yet it failed to protect the fish against bacterial challenges and did not enhance growth. In conclusion, although *E. globulus* PE demonstrates potential as an immunostimulant in aquaculture, further research is essential to identify the specific volatile compounds responsible for its antimicrobial and immunomodulatory effects. Additionally, strategies to preserve these bioactive components during feed preparation and storage should be explored, as well as the efficacy of PE against a broader range of aquatic pathogens.

## Figures and Tables

**Table 1:** Bacterial species used for MIC and MBC using *E. globulus* PE.

Bacterial Strains	Collection Number	Code	Year
*Aeromonas hydrophila*	17410018	INCQS 00318	2014
*Aeromonas jandaei*	17410036	LHZ-FZEA-USP#01/19	2019
*Escherichia coli*	17410013	INCQS 00171	2013
*Plesiomonas shigelloides*	17410038	LHZ-FZEA-USP#03/19	2019
*Pseudomonas aeruginosa*	17410032	ATCC 00313	2018
*Salmonella enteritidis*	17410010	ATCC 13076	2009
*Streptococcus agalactiae*	17410028	INCQS 00128	2016

**Table 2:** MIC and MBC results of six bacterial strains isolated or not from fish exposed to *E. globulus* pyroligneous extract (PE).

Bacterial Strains	MIC (%)	MBC (%)
*Aeromonas hydrophila*	6.25	12.5
*Aeromonas jandaei*	6.25	12.5
*Escherichia coli*	6.25	12.5
*Plesiomonas shigelloides*	3.12	-
*Pseudomonas aeruginosa*	6.25	12.5
*Salmonella enteritidis*	6.25	12.5
*Streptococcus agalactiae*	6.25	12.5

**Table 3:** Productive performance of *O. niloticus* fed with 0.5 and 1% of *E. globulus* pyroligneous extract (PE) for 30 days.

Treatments	IIW	FIW	IL	FL	Cons	WG	MWG	FCR	MDWG	Growth	K
Control	13.7 ± 1.1	48.2 ± 3.7	9.5 ± 0.4	13.2 ± 0.3	915.7 ± 84.5	1036.6 ± 79.9	34.5 ± 2.6	0.88 ± 0.01	1.33 ± 0.1	3.7 ± 0.5	2.07 ± 0.01
0.5%	12.7 ± 0.5	41.8 ± 0.9	8.6 ± 0.1	12.3 ± 0.1	868 ± 22.8	874 ± 13.1	29.1 ± 0.4	0.99 ± 0.02	1.12 ± 0.01	3.7 ± 0.1	2.20 ± 0.04
1%	11.6 ± 0.3	40.8 ± 1.2	8.3 ± 0.1	12.4 ± 0.1	1002 ± 38.8	875.6 ± 36.2	29.2 ± 1.2	1.15 ± 0.09	1.12 ± 0.04	4.1 ± 0.1	2.11 ± 0.03
*p*-Value	0.4630	0.3530	0.1780	0.2650	0.4830	0.3120	0.3120	0.1810	0.3000	0.8660	0.2650

IIW: initial individual weight; FIW: final individual weight; IL: initial length; FL: final length; Cons: feed consumption; WG: weight gain; MWG: mean weight gain; FCR: feed conversion ratio; MDWG: mean daily weight gain; K: condition factor.

## Data Availability

The original contributions presented in this study are included in the article. Further inquiries can be directed to the corresponding author.

## References

[1] FAO (2022). The State of World Fisheries and Aquaculture 2022.

[2] Mian G.F., Godoy D.T., Leal C.A.G., Yuhara T.Y., Costa G.M., Figueiredo H.C.P. (2009). Aspects of the natural history and virulence of *S. agalactiae* infection in Nile tilapia. Vet. Microbiol..

[3] Pankhurst N.W., Van Der Kraak G. (1997). Effects of stress on reproduction and growth in fish. Fish Stress Health Aquac..

[4] Alderman D.J., Hastings T.S. (1998). Antibiotic use in aquaculture: Development of antibiotic resistance—Potential for consumer health risks. Int. J. Food Sci. Technol..

[5] Migliore L., Civitareale C., Brambilla G., Dojmi Di Delupis G. (1997). Toxicity of several important agricultural antibiotics to Artemia. Water Res..

[6] Ahmad Pauzi N., Mohamad N., Azzam-Sayuti M., Md Yasin I.S., Saad M.Z., Nasruddin N.S., Azmai M.N.A. (2020). Antibiotic susceptibility and pathogenicity of *Aeromonas hydrophila* isolated from red hybrid tilapia (*Oreochromis niloticus*×*Oreochromis mossambicus*) in Malaysia. Vet. World..

[7] Witte W. (1998). Medical consequences of antibiotics use in agriculture. Science.

[8] Xiong W., Sun Y., Zhang T., Ding X., Li Y., Wang M., Zeng Z. (2015). Antibiotics, Antibiotic Resistance Genes, and Bacterial Community Composition in Fresh Water Aquaculture Environment in China. Microb. Ecol..

[9] Ebrahimi G., Ouraji H., Khalesi M.K., Sudagar M., Barari A., Dangesaraki M.Z., Khalili K.H. (2012). Effects of a prebiotic, Immunogen^®^, on feed utilization, body composition, immunity and resistance to *Aeromonas hydrophila* infection in the common carp *Cyprinus carpio* (Linnaeus) fingerlings. J. Anim. Physiol. Anim. Nutr..

[10] Munir M.B., Hashim R., Chai Y.H., Marsh T.L., Mohd Nor S.A. (2016). Dietary prebiotics and probiotics influence growth performance, nutrient digestibility and the expression of immune regulatory genes in snakehead (*Channa striata*) fingerlings. Aquaculture.

[11] Giri S.S., Sukumaran V. (2013). Potential probiotic *Lactobacillus plantarum* VSG3 improves the growth, immunity, and disease resistance of tropical freshwater fish, Labeo rohita. Fish Shellfish. Immunol..

[12] Nguyen T.L., Park C.-I., Kim D.-H. (2017). Improved growth rate and disease resistance in olive flounder, *Paralichthys olivaceus*, by probiotic *Lactococcus lactis* WFLU12 isolated from wild marine fish. Aquaculture.

[13] Tan H.Y., Chen S.-W., Hu S.-Y. (2019). Improvements in the growth performance, immunity, disease resistance, and gut microbiota by the probiotic *Rummeliibacillus stabekisii* in Nile tilapia (*Oreochromis niloticus*). Fish Shellfish. Immunol..

[14] Zhang R., Wang X.W., Liu L.L., Cao Y.C., Zhu H. (2020). Dietary oregano essential oil improved the immune response, activity of digestive enzymes, and intestinal microbiota of the koi carp, *Cyprinus carpio*. Aquaculture.

[15] Zheng Z.L., Tan J.Y.W., Liu H.Y., Zhou X.H., Xiang X., Wang K.Y. (2009). Evaluation of oregano essential oil (*Origanum heracleoticum* L.) on growth, antioxidant effect and resistance against *Aeromonas hydrophila* in channel catfish (*Ictalurus punctatus*). Aquaculture.

[16] Almeida R.S.R., Taccini M.M., Moura L.F., Ceribelli U.L., Brito J.O., Gloria E.M. (2019). Potential of Pyroligneous Extract of Eucalyptus Wood as a Preservative of Cosmetic and Sanitizing Products. Waste Biomass Valorization.

[17] de Moraes França Ferreira P., da Silva Nascimento L., Coelho Dias D., da Veiga Moreira D.M., Lúcia Salaro A., Duca de Freitas M.B., Souza Carneiro A.P., Sampaio Zuanon J.A. (2014). Essential Oregano Oil as a Growth Promoter for the Yellowtail Tetra, Astyanax Altiparanae. J. World Aquac. Soc..

[18] Pereira E.G., Fauller H., Magalhães M., Guirardi B., Martins M.A. (2022). Potential use of wood pyrolysis coproducts: A review. Environ. Prog. Sustain. Energy.

[19] Bektaş S., Murat Ö. (2022). Antibacterial activity of eucalyptus (*Eucalyptus camaldulensis*) essential oil against fish pathogen bacterium, *Aeromonas caviae*. Mar. Sci. Tech. Bull..

[20] Vaseeharan B., Thaya R. (2014). Medicinal plant derivatives as immunostimulants: An alternative to chemotherapeutics and antibiotics in aquaculture. Aquac. Int..

[21] Mohamadi M., Zamini A.A., Vahabzadeh H. (2013). Evaluation of Antibacterial Properties of *Eucalyptus spp* and *Plelargonium roseum* Extracts in Common carp, *Cyprinus carpio* and Their Effectson Blood Indices. Mid-East J. Sci. Res..

[22] Elumalai P., Kurian A., Lakshmi S., Faggio C., Esteban M.A., Ringø E. (2020). Herbal Immunomodulators in Aquaculture. Rev. Fish. Sci. Aquac..

[23] Reverter M., Bontemps N., Lecchini D., Banaigs B., Sasal P. (2014). Use of plant extracts in fish aquaculture as an alternative to chemotherapy: Current status and future perspectives. Aquaculture.

[24] Van Hai N. (2015). The use of medicinal plants as immunostimulants in aquaculture: A review. Aquaculture.

[25] Souza J.B.G., Ré-Poppi N., Raposo J.L. (2012). Characterization of pyroligneous acid used in agriculture by gas chromatography-mass spectrometry. J. Braz. Chem. Soc..

[26] Grewal A., Gunupuru L.R. (2018). Production, prospects and potential application of pyroligneous acid in agriculture. J. Anal. Appl. Pyrolysis.

[27] Dawood M.A.O., El Basuini M.F., Yilmaz S., Abdel-Latif H.M.R., Alagawany M., Abdul Kari Z.A., Razab M.K.A.A., Hamid N.K.A., Moonmanee T., Doan H.V. (2022). Exploring the Roles of Dietary Herbal Essential Oils in Aquaculture: A Review. Animals.

[28] Sheikhzadeh N., Soltani M., Ebrahimzadeh-Mousavi H.A., Shahbazian N., Norouzi M. (2011). Effects of *Zataria multiflora* and *Eucalyptus globolus* essential oils on haematological parameters and respiratory burst activity in *Cyprinus carpio*. Iran J. Fish Sci..

[29] Sivaraj A., Devagnanaroopan J., Palani B., Senthilkumar B. (2017). View of Antibacterial activity of *Eucalyptus oblique* aqueous leaf extract on selective *Vibrio* species of *Penaeus monodon* culture hatchery. World J. Pharm. Sci..

[30] Zarei S., Bahador N., Mirbakhsh M., Pazir M.K. (2019). Antibacterial activity of aqueous extract of *Eucalyptus camaldulensis* in different salinity and temperature against *Vibrio harveyi* (PTCC1755) and *Vibrio alginolyticus* (MK641453.1). J. Pharmacogn. Phytochem..

[31] Ghalem B.R., Mohamed B. (2008). Antibacterial activity of leaf essential oils of Eucalyptus globulus and Eucalyptus camaldulensis. Afr. J. Pharm. Pharmacol..

[32] Gholipourkanani H., Buller N., Lymbery A. (2019). In vitro antibacterial activity of four nano-encapsulated herbal essential oils against three bacterial fish pathogens. Aquac. Res..

[33] Harkat-Madouri L., Asma B., Madani K., Bey-Ould Si Said Z., Rigou P., Grenier D., Allalou H., Remini H., Adjaoud A., Boulekbache-Makhlouf L. (2015). Chemical composition, antibacterial and antioxidant activities of essential oil of *Eucalyptus globulus* from Algeria. Ind. Crops Prod..

[34] Park J.W., Wendt M., Heo G.-J. (2016). Antimicrobial activity of essential oil of *Eucalyptus globulus* against fish pathogenic bacteria. Lab. Anim. Res..

[35] FAO (2024). The State of World Fisheries and Aquaculture 2024.

[36] PeixeBR, Anuário 2025—PeixeBR, (2025). https://www.peixebr.com.br/anuario-2025/.

[37] Ho S.P., Hsu T.Y., Chen M.H., Wang W.S. (2000). Antibacterial effect of chloramphenicol, thiamphenicol and florfenicol against aquatic animal bacteria. J. Vet. Med. Sci..

[38] Ulkhaq M.F., Budi D.S., Rahayu N.N. (2020). The effect of temperature, salinity and antimicrobial agent on growth and viability of *Aeromonas hydrophila*. IOP Conf. Ser. Earth Environ. Sci..

[39] Dairiki J.K., Majolo C., Chagas E.C., Chaves F.C.M., de Oliveira M.R., Morais I.D.S. (2013). Procedimento para Inclusão de óleos Essenciais em rações para Peixes. https://www.sidalc.net/search/Record/dig-infoteca-e-doc-973005/Description.

[40] Baba E., Acar Ü., Öntaş C., Kesbiç O.S., Yılmaz S. (2016). Evaluation of *Citrus limon* peels essential oil on growth performance, immune response of Mozambique tilapia *Oreochromis mossambicus* challenged with *Edwardsiella tarda*. Aquaculture.

[41] Brum A., Pereira S.A., Owatari M.S., Chagas E.C., Chaves F.C.M., Mouriño J.L.P., Martins M.L.M. (2017). Effect of dietary essential oils of clove basil and ginger on Nile tilapia (*Oreochromis niloticus*) following challenge with *Streptococcus agalactiae*. Aquaculture.

[42] Firmino J.P., Galindo-Villegas J., Reyes-López F.E., Gisbert E. (2021). Phytogenic bioactive compounds shape fish mucosal immunity. Front. Immunol..

[43] Ranzani-Paiva M.J.T., Pádua S.B., Tavares-Dias M., Egami M.I. (2013). Métodos para Análise Hematológica em Peixes.

[44] Levy-Pereira N., Yasui G.S., Evangelista M.M., Nascimento N.F., Santos M.P., Siqueira-Silva D.H., Monzani P.S., Senhorini J.A., Pilarski F. (2019). In vivo phagocytosis and hematology in *Astyanax altiparanae*, a potential model for surrogate technology. Braz. J. Biol..

[45] da Silva B.A., Feijó F.M.C., Alves N.D., Pimenta A.S., Benicio L.D.M., Silva-Júnior E.C., Santos C.S., Pereira A.F., Moura Y.B.F., Gama G.S.P. (2023). Use of a product based on wood vinegar of *Eucalyptus* clone I144 used in the control of bovine mastitis. Vet. Microbiol..

[46] de Souza J.L.S., Alves T., Camerini L., Nedel F., Campos A.D., Lund R.G. (2021). Antimicrobial and cytotoxic capacity of pyroligneous extracts films of *Eucalyptus grandis* and chitosan for oral applications. Sci. Rep..

[47] de Souza Araújo E., Pimenta A.S., Feijó F.M.C., Castro R.V.O., Fasciotti M., Monteiro T.V.C., de Lima K.M.G. (2018). Antibacterial and antifungal activities of pyroligneous acid from wood of *Eucalyptus urograndis* and *Mimosa tenuiflora*. J. Appl. Microbiol..

[48] Agency for Toxic Substances and Disease Registry (ATSDR) (2008). Toxicological profile for phenol.

[49] Agency for Toxic Substances and Disease Registry (ATSDR) (2008). Toxicological Profile for Cresols.

[50] Pimenta A.S., Fasciotti M., Monteiro T.V.C., Lima K.M.G. (2018). Chemical Composition of Pyroligneous Acid Obtained from Eucalyptus GG100 Clone. Molecules.

[51] Nurudeen N.D., Ayisi C.L., Ampofo-Yeboah A. (2022). Effects of *Eucalyptus globulus* leaf extract on growth performance, feed utilization and blood biochemistry of Nile tilapia, *Oreochromis niloticus*. Veterinaria.

[52] Hoseini S.M., Mirghaed A.T., Iri Y., Ghelichpour M. (2018). Effects of dietary cineole administration on growth performance, hematological and biochemical parameters of rainbow trout (*Oncorhynchus mykiss*). Aquaculture.

[53] Peterfalvi A., Miko E., Nagy T., Reger B., Simon D., Miseta A., Czéh B., Szereday L. (2019). Much More Than a Pleasant Scent: A Review on Essential Oils Supporting the Immune System. Molecules.

[54] Awad E., Awaad A. (2017). Role of medicinal plants on growth performance and immune status in fish. Fish. Shell. Immunol..

[55] Immanuel G., Uma R.P., Iyapparaj P., Citarasu T., Peter S.M.P., Babu M.M., Palavesam A. (2009). Dietary medicinal plant extracts improve growth, immune activity and survival of tilapia *Oreochromis mossambicus*. J. Fish. Biol..

[56] Reverter M., Tapissier-Bontemps N., Sarter S., Sasal P., Caruso D. (2021). Moving towards more sustainable aquaculture practices: A meta-analysis on the potential of plant-enriched diets to improve fish growth, immunity and disease resistance. Rev. Aquac..

[57] Lin Z., An S., Zhou C., Chen Y., Gao Z., Feng J., Lin H., Xun P., Yu W. (2025). Effects of eucalyptus essential oil on growth, immunological indicators, disease resistance, intestinal morphology and gut microbiota in Trachinotus ovatus. Microorganisms.

